# Developing a Learning Progression for Probability Based on the GDINA Model in China

**DOI:** 10.3389/fpsyg.2020.569852

**Published:** 2020-09-23

**Authors:** Shengnan Bai

**Affiliations:** School of Mathematics and Statistics, Northeast Normal University, Changchun, China

**Keywords:** probability, learning progression, GDINA model, attribute hierarchy, learning pathway

## Abstract

This research focuses on developing a learning progression of probability for middle school students, and it applies the GDINA model in cognitive diagnosis models to data analysis. GDINA model analysis firstly extracted nine cognitive attributes and constructed their attribute hierarchy and the hypothesized learning progression according to previous studies, curriculum standards, and textbooks. Then the cognitive diagnostic test was developed based on Q-matrix theory. Finally, we used the GDINA model to analyze a sample of 1624 Chinese middle school students’ item response patterns to identify their attribute master patterns, verify and modify the hypothesized learning progression. The results show that, first of all, the psychometric quality of the measurement instrument is good. Secondly, the hypothesized learning progression is basically reasonable and modified according to the attribute mastery probability. The results also show that the level of probabilistic thinking of middle school students is improving steadily. However, the students in grade 8 are slightly regressive. These results demonstrate the feasibility and superiority of using cognitive diagnosis models to develop a learning progression.

## Introduction

Learning progression is defined as ‘descriptions of the successively more sophisticated ways of thinking about a topic that can follow one another as children learn about and investigate a topic over a broad span of time (e.g., 3–5 years)’ (National Research Council, 2007). Although different perspectives of concern formed different definitions of learning progression (Catley et al., 2005; Duncan and Cindy, 2009; Mohan et al., 2009), they all focus on the study of core knowledge to investigate students’ cognitive development process. It seems that learning progression is an important channel for the dialogue among theoretical researchers, curriculum planners, educational decision-makers and exam examiners, a bridge between learning research and classroom teaching, and a tool with the most potential to connect curriculum standards, teaching and evaluation and promote the consistency of the three.

Quantitative analysis plays an essential part in developing a learning progression. The initial research on learning progression was built on descriptive statistical results. At present, the most effective and widely used method is Rasch measurement theory (Liu and Collard, 2005; Liu and McKeough, 2005; Johnson, 2013; Todd and Romine, 2016), which estimates the item difficulty parameter as the same level as the students’ ability parameter (Rasch, 1960/1980). Rasch analysis assumes unidimensionality, that is, a single trait affects the responses of the participants (Wilson, 2005; Chen et al., 2017a). However, because the core concept covers a wide range of attributes, it is difficult to strictly satisfied the unidimensionality assumption in practice. The learning progression constructed by the above two quantitative analysis methods is a linear step-by-step development process of students as they increase in grade or as time goes by, and the ability level of students is estimated mainly through the total score of the test.

Since the core concepts are directly related to the internal logical structure of the discipline, they are not all linear, so students can understand core concepts through different learning pathways (Alonzo and Steedle, 2008). In recent years, the research on the learning progression of core concepts has been integrated into the process of individual cognitive structure gradually becoming complete. Since the beginning of the last century, psychometrics and cognitive psychology have been increasingly dissatisfied with assessing the ability level of the individual from macro perspective, so a new generation of psychometrics theory has developed a cognitive diagnosis model for the purpose of diagnosing students’ cognitive process, processing skills or knowledge structure. Therefore, researchers began to use it as a quantitative analysis method to provide technical support for the construction of learning progression evaluation system, so as to deeply evaluate student’ knowledge structure (Derek and Alonzo, 2012; Chen et al., 2017b; Gao et al., 2017).

Compared with traditional methods, cognitive diagnosis models have the following advantages. First, cognitive diagnosis models directly integrate cognitive variables to estimate the attribute mastery pattern (AMP) of each student, thus realizing the measurement and evaluation of individual’s cognitive level from the micro perspective. Second, the attributes that students have and have not mastered can be identified from their responses to the test items. These attributes are distributed at different levels of learning progression, which helps to verify and modify the hypothesized learning progression. Third, it is beneficial to promote personalized education. Each level of learning progression based on the cognitive diagnosis models has multiple AMPs, that is, there are multiple learning pathways from the low level to high level, so as to provide targeted teaching according to the individual student’s AMP. Generalized Deterministic Inputs, Noisy and Gate (GDINA) model (de la Torre, 2011), as a saturated cognitive diagnosis model, breaks through the assumptions of the previous simplified cognitive diagnosis models on attribute action mechanism, making the model more flexible and widely used. Whereas, there are few studies have been done on learning progression based on the GDINA model.

As one of the most basic core qualities throughout the mathematics curriculum, probability literacy has now become an indispensable quality for every citizen to enter the society (Scheaffer, 1984; Biehler, 1994; Aitken, 2009). However, studies have repeatedly shown that students always have different degrees of cognitive difficulties in the development of probabilistic thinking. Jones et al. (1997, 1999) proposed a framework to describe students’ cognition of probability, in which students’ understanding of probability concepts is divided into subjective level, transitional level, informal quantitative level and numerical level. English, Fischbein and Lecoutre found that students cannot naturally understand the sample space, because the basic results in different orders should be distinguished and counted as different results (Fischbein and Gazit, 1984; Lecoutre et al., 1990; English, 1993). Whereas, further analysis shows that although previous research on probability investigated all knowledge points, they did not pay enough attention to the core knowledge. Thus, the introduction of learning progression provides a new research perspective for probability.

As shown in the above literature review, from the perspective of students, there are many stubborn misunderstandings and preconceptions in the learning of probability concepts (Green, 1982; Fischbein and Gazit, 1984; Fischbein et al., 1991; Williams and Amir, 1995; Moritz et al., 1996; Potyka and Thimm, 2015). However, Liu and Thompson’s research provided a rich description of the kinds of difficulties experienced by teachers in developing coherent and powerful understandings of probability (Liu and Thompson, 2007). From the perspective of empirical research, the existing studies on learning progression ignored the establishment of a cognitive model, so the probabilistic cognitive structure of individual students cannot be systematically described. Additionally, the nature of cognitive diagnosis and learning progression is very consistent, so using it as a measurement tool to construct the learning progression of probability is well worth further exploration.

To address the issues already outlined and to begin to fill the gaps in the previous research, the present study attempts to: (a) judge whether developed measurement instrument is appropriate to evaluate learning progression of students’ probability; (b) verify and modify the hypothesized learning progression by the results of the GDINA model analysis; (c) identify what levels of students’ AMPs and provide proper learning pathways accordingly.

## Hypothesized Learning Progression

The current course distribution of probability concept is as follows: intuitive perception of probability concepts through experiments, games and other activities is arranged in grades 4 to 6. The systematic study of preliminary probability is set in grade 9, which is mainly the teaching of one-dimensional probability concepts. Further probability knowledge is arranged in grade 11. The curriculum goals are to deeply learn two-dimensional probability concepts and to preliminarily understand relevant probability concepts of finite dimensions. On this basis, the attribute selection, the attribute hierarchy and the hypothesized learning progression are studied one by one.

### Attribute Selection

According to the basic process of cognitive diagnosis, the cognitive attributes contained in probability should be extracted first (Tatsuoka, 2009; de la Torre, 2011; Basokcu, 2014; Rupp and van Rijn, 2018). The most common probability concepts in previous research were the following: randomness, sample space, probability of an event, probability comparisons (Fischbein, 1975; Biggs and Collis, 1982; Liu and Zhang, 1985; Jones et al., 1997, 1999; Li, 2003; Piaget and Inhelder, 2014; He and Gong, 2017). Other studies have also explored students’ ability to make probability estimation (Acredolo et al., 1989). However, the components mentioned above do not explicitly indicate the impact of dimensions.

Taking into account previous studies, curriculum standards and textbooks, students’ understanding of one-dimensional probability concepts and two-dimensional probability concepts is not synchronized (Liu and Zhang, 1985; Jones et al., 1997; Li, 2003). Hence, when identifying cognitive attributes, the probability was not only divided into randomness, sample space, probability of an event, probability comparisons and probability estimation, but also the effect of dimension was considered. Consequently, we obtained the nine cognitive attributes of probability as follows.

A1: Randomness: distinguish between certain events, random events, and impossible events.
A2: One-dimensional sample space: list all possible outcomes of a one-dimensional event.
A3: Two-dimensional sample space: list all possible outcomes of a two-dimensional event.
A4: One-dimensional probability comparisons: compare the probability of one-dimensional events.
A5: Two-dimensional probability comparisons: compare the probability of two-dimensional events.
A6: Probability of a one-dimensional event: calculate the probability of a one-dimensional event by definition.
A7: Probability of a two-dimensional event: calculate the probability of a two-dimensional event by definition.
A8: Probability estimation of a one-dimensional event: estimate the probability of a one-dimensional event by frequency.
A9: Probability estimation of a two-dimensional event: estimate the probability of a two-dimensional event by frequency.

### Attribute Hierarchy

On the basis of attributes selected before, the attribute hierarchy was constructed by considering previous studies and the curricular sequences of the relevant probabilistic content in the curriculum standards and textbooks. Some studies suggested that the understanding of randomness is the starting point for probabilistic thinking, and this ability increases with age (Williams and Amir, 1995; Chan, 1997; Jones et al., 1997). This indicates that randomness is a precondition of sample space and probability estimation. Furthermore, the understanding of sample space is central to understanding probability (Van de Walle et al., 2016). He and Gong (2017) found that students aged 6 to 14 must master the sample space in order to perform well in calculating the probability of an event by definition. Zhang’s team demonstrated that students’ understanding of the sample space is superior than probability comparisons (Zhang et al., 1985). This indicates that sample space is a premise of probability of an event and probability comparisons.

**FIGURE 1:**
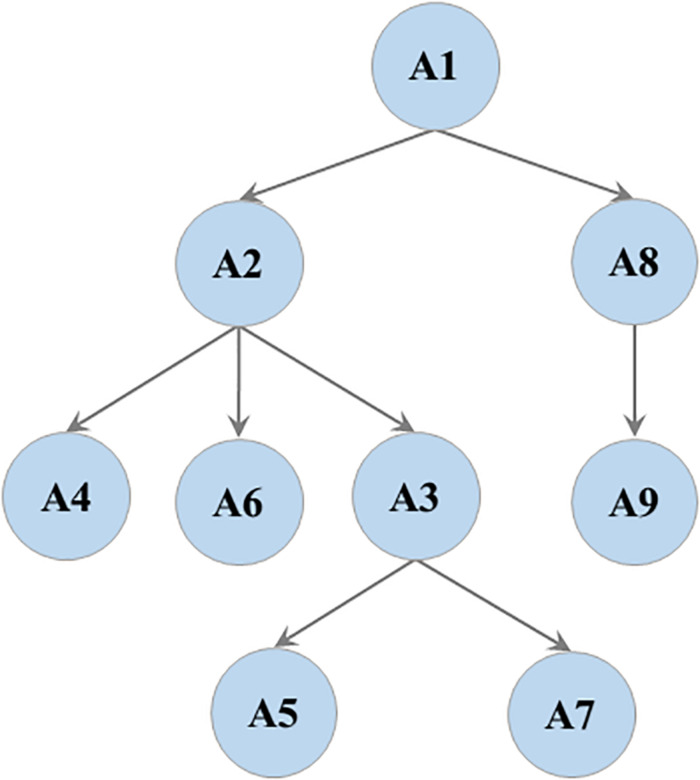
The attribute hierarchy.

Considering the impact of dimensions on students’ understanding of probability, students who can consistently list all possible outcomes of a one-dimensional event were often inconsistent or unsystematic in listing all possible outcomes of a two-dimensional event (Liu and Zhang, 1985; Jones et al., 1997; He and Gong, 2017). Moreover, probability estimation is an intuitive way to understand the probability of an event through a large number of repeated experiments. Chapin et al. (2003) argued that students in grades 3 to 5 can initially understand the relationship between the frequency and probability of a one-dimensional event. However, interviews with middle school teachers revealed that students also made some errors in estimating the probability of a two-dimensional event, indicating that probability estimation of a one-dimensional event is the prerequisite of probability estimation of a two-dimensional event. As such, the attribute hierarchy was constructed (Figure 1). Thereafter, the attribute hierarchy was tested through mathematics curriculum standards, mathematics textbooks, and interviews with teachers. Attribute hierarchy was found to be basically consistent with the curricular sequences and instructional sequences of the related mathematical topics.

### Hypothesized Learning Progression

In light of the above analysis, we developed the hypothesized learning progression of probability for middle school students relied on the previous studies, curriculum standards and textbooks. Considering the influence brought by the dimensions, students’ understanding of probability was investigated from five aspects: randomness, sample space, probability of an event, probability comparisons and probability estimation. We then used the SOLO (Structure of the Observed Learning Outcome) taxonomy that developed from Piaget’s cognitive development phase theory to clarify the learning progression levels (Biggs and Collis, 1982, 1991).

In the hypothesized learning progression of probability (see Table 1), Level 1 does not involve any attributes of probability, indicating that the probabilistic thinking of students at Level 1 has not yet begun to develop. When the students reach Level 2, students begin to understand the one-dimensional probability concepts, indicating that they have mastered at least one of A1, A2, A4, A6, and A8. On the basis of Level 2, students at Level 3 can perform two-dimensional sample space and probability estimation of a two-dimensional event, indicating that students’ mastery of A3 and A9. At last, when students reach Level 4, they can understand two-dimensional probability comparisons and probability of a two-dimensional event. This indicates that students have mastered all attributes of probability. So far, we established the correspondence between the hypothesized learning progression levels and the attributes of probability, which will help to verify and modify the hypothesized learning progression through the analysis of GDINA model.

**TABLE 1:** Hypothesized learning progression of probability.

Level	Content	Attributes
1	Students cannot master any attributes related to probability.	None
2	Students begin to understand the one-dimensional probability concepts, but they cannot transfer their understanding of one-dimensional probability concepts to two-dimensional probability concepts.	At least one of A1, A2, A4, A6, and A8
3	Students can perform two-dimensional sample space and probability estimation of a two-dimensional event.	Further master at least one of A3 and A9
4	Students can understand two-dimensional probability comparisons and probability of a two-dimensional event. Furthermore, they can build a connection between one-dimensional probability concepts and two-dimensional probability concepts.	Further master A5 and A7

## Materials and Methods

### Item Design

The Q-matrix (Table 2), which is established by the selected attributes and their attribute hierarchy, presented the correspondence between each item and each attribute and was used to guide the item design. Q-matrix is based on the design principles proposed by Tu et al. (2012). The first is that the item assessment patterns should include the Reachability Matrix. The second is that each attribute is measured no less than three times. In the Q-matrix, ‘1’ means that the attribute is measured in this item, and ‘0’ means that the attribute is not measured in this item. For instance, ‘100000000’ means that item 1, item 2, item 3, and item 4 only measure A1, and ‘100000011’ means that item 24, item 25 and item 26 measure A1, A8, and A9.

**TABLE 2:** Q-matrix of attributes and items.

	Attribute								
Item	A1	A2	A3	A4	A5	A6	A7	A8	A9
1	1	0	0	0	0	0	0	0	0
2	1	0	0	0	0	0	0	0	0
3	1	0	0	0	0	0	0	0	0
4	1	0	0	0	0	0	0	0	0
5	1	1	0	0	0	0	0	0	0
6	1	1	0	1	0	0	0	0	0
7	1	1	0	0	0	0	0	0	0
8	1	1	0	1	0	0	0	0	0
9	1	1	0	0	0	0	0	0	0
10	1	1	0	1	0	0	0	0	0
11	1	1	0	0	0	1	0	0	0
12	1	1	0	0	0	1	0	0	0
13	1	1	0	0	0	1	0	0	0
14	1	1	1	0	0	0	0	0	0
15	1	1	1	0	1	0	0	0	0
16	1	1	1	0	0	0	0	0	0
17	1	1	1	0	1	0	0	0	0
18	1	1	1	0	0	0	0	0	0
19	1	1	1	0	1	0	0	0	0
20	1	1	1	0	0	0	1	0	0
21	1	1	1	0	0	0	1	0	0
22	1	1	1	0	0	0	1	0	0
23	1	0	0	0	0	0	0	1	0
24	1	0	0	0	0	0	0	1	1
25	1	0	0	0	0	0	0	1	1
26	1	0	0	0	0	0	0	1	1

Five mathematics teachers, two subject experts, two mathematics educators and two psychometricians were invited to develop the instrument. The mathematics teachers came from key middle schools in Fujian, Shanxi, Henan and Inner Mongolia, as well as a teaching and research staff from Qinghai Province. The subject experts consisted of a professor and an associate professor who study probability and statistics. The mathematics educators were composed of two professors engaged in mathematics education research. The psychometricians were comprised of a professor and a Ph.D. candidate who do research in psychometrics.

Based on the Q-matrix, curriculum standards and textbooks, the study developed a cognitive diagnostic test of probability, which consists of 26 items and each item corresponds to a specific item assessment pattern (IAP). All items are in multiple-choice or short-answer format. All items are dichotomous, with the correct score of ‘1’ and the wrong score of ‘0.’ Table 3 presents some example items and their corresponding IAPs.

**TABLE 3:** Example items from the probability test.

Item number	Content	IAP
1	Roll a fair dice and the number rolled is greater than 6. Please determine the type of this event. (A) Certain (B) Random (C) Impossible	100000000
7	Randomly select a number from the set {1, 2, 3, 4, 5}. Please write out how many possible outcomes there are.	110000000
18	Roll two fair dice and observe the number on the up side. Please list all possible outcomes of the numbers rolled by the two dice.	111000000

### Participants and Procedure

According to the level of economic development, mainland China can be divided into four types: the most developed areas, the developed areas, the moderately developed areas and the underdeveloped areas. Since the moderately developed areas cover 23 provinces and cities, accounting for a large part of the mainland (Xie and Lu, 2011), the schools and corresponding students in these areas were selected in this study.

In the end, six junior high schools and five high schools were selected from the moderately developed areas. A total of 1624 students participated in this study (Table 4). To ease the tension of the students during the test, we informed them that their test results will not affect their academic rankings this semester. The time allocated to the test was 40 min.

**TABLE 4:** Structure description of the sample.

Grade	7	8	9	10	11
Total number	323	333	302	354	312

### Data Analysis

#### Instrument Functioning

Parscale 4.1, R and SPSS 22.0 were used to investigate the psychometric quality of the developed measurement instrument. First, the rationality of attribute selection and the attribute hierarchy should be attested. Specifically, we performed a linear regression analysis to see if the attributes measured by the item can predict the item difficulty level. We used the hierarchy consistency index (HCI) to measure the degree of matching between the actual item response pattern (IRP) and the expected response pattern under the attribute hierarchy. Second, the test reliability and test validity should be explored. Attribute test–retest reliability was used as the test reliability measure under Cognitive Diagnosis Theory, indicating the internal consistency of each attribute (Templin and Bradshaw, 2013). As for test validity, since our study used a cognitive diagnosis model, the identifiability of the Q-matrix was used as evidence of the test validity. Third, the quality of each item should be explored. This includes the examination of item fitting index, item difficulty and item discrimination. In addition, students with abnormal responses were identified and analyzed by participant fitting index.

#### GDINA Model Analysis

In cognitive diagnosis assessment, the ability of each student is presented as AMP (attribute master pattern). Attribute refers to the knowledge, skills and strategies required for a student to correctly complete a test item. AMP is a description of whether a student has mastered each attribute. Where, ‘1’ means that the attribute is mastered, and ‘0’ means that the attribute is not mastered.

The GDINA model was used to classify students into different AMPs represented by the observed IRPs. First, the rationality of attribute selection and attribute hierarchy should be verified. Then, the identifiability of Q-matrix and the psychometric quality of the cognitive diagnostic test must be judged. Finally, student’s AMP was estimated from his or her IRP through the classical estimation method. Ideally, a student should only correctly answer items that measure the attributes he or she mastered, and incorrectly answer items that measure at least one attribute that he or she did not master. For more information about the GDINA model estimation program, please refer to de la Torre (2011). The above analysis was performed using the GDINA model program in the R package (CDM package). The item response function of the GDINA model is as follows:

P(Xij=1|αιj*)=δj0+∑k=1Kj*δjkαιk+∑k=′k+1Kj*∑k=1Kj*-1δjkk′αιkαιk′+⋯+δj12⋯⋅Kj*∏k=1Kj*αιk

The function above can be decomposed into the sum of the effects due to the presence of specific attributes and all their possible interactions. δ_*j0*_ is the intercept of item *j*, called the baseline probability, that is, the probability that the participant answers the item correctly without mastering all the attributes measured by this item. The value is a non-negative value and can be regarded as the guessing parameter. δ_*jk*_ is the main effect of attribute *k* on item *j*, which is generally a non-negative value. It represents the effect of increasing the probability of answering this item correctly because the participant has mastered the attribute *k*. The larger the value, the greater the contribution of mastering the attribute to the correct item *j*. δ_*jkk’*_ is the interaction effect of attribute *k* and attribute *k*′ on item *j*. $\delta_{j12\cdot \cdot \cdot \cdot K_{j}^{*}}$ measures the interaction effect between all attributes for item *j*.

#### Learning Progression Verification and Modification

Due to the correspondence between the hypothesized learning progression levels and the attributes contained in probability presented in Table 1, the attribute mastery probability analyzed by the GDINA model was used to verify and modify the hypothesized learning progression. Students are expected to develop a successively more sophisticated understanding of probability based on the hypothesized learning progression levels.

First, students will master the attributes regarding the one-dimensional probability concepts. Then, students will enter the initial stage of two-dimensional probabilistic thinking, that is, they will continue to learn the sample space and compare the probability of two-dimensional events. It ends with students being able to build a connection between one-dimensional probability concepts and two-dimensional probability concepts. If the hypothesized learning progression is reasonable, the attributes at higher levels are generally more difficult to master than the attributes at lower levels.

## Results

### Instrument Functioning

In this study, a cognitive diagnostic test was developed under the guidance of the GDINA model. Tu et al. (2012) suggested to first attest the rationality of the attribute selection and the attribute hierarchy. For attribute selection, the result of linear regression analysis with the item difficulty as the dependent variable and the columns of the Q-matrix as the independent variables shows that the adjusted *R*^2^ value is 0.875. This means the explanatory power of the selected attributes to the item difficulty is 87.5%, which verifies the attribute selection. For attribute hierarchy, Cui and Leighton (2009) proposed that it is feasible to use HCI index to test the rationality of attribute hierarchy. Wang and Gierl (2007) pointed out that if the mean value of HCI index is greater than 0.6, the attribute hierarchy has good rationality. Based on the current data, the mean value of HCI index is 0.90, which proves that the attribute hierarchy is reasonable.

Regarding the quality of cognitive diagnostic test, the reliability and validity needs to be checked. Based on attribute test–retest reliability, the internal consistency value of each attribute ranges from 0.88 to 0.99, indicating that each attribute has good reliability. Then the test was prepared according to the design principles of Q-matrix proposed by Tu et al. (2012), which can confirm the validity of the test.

As for the quality of each item, the item fitting index RMSEA for all items is less than 0.08, with an average of 0.03, indicating that each item has a good fit to GDINA model. The item difficulty index under CTT shows that the difficulty value of most items is between 0.37 and 0.84, with an average of 0.62, and only seven items have difficulty values higher than 0.84. The estimation of item difficulty under IRT shows that the difficulty range is between −3.41 and 0.95. As for the item discrimination, when the discrimination is greater than 0.4, the item is considered excellent (Ray and Margaret, 2003; Tu et al., 2019), and all items meet the standard.

According to the participant fitting index, if the index is greater than −2, the participant’s response is in good agreement with the model. In this study, 94.6% of the students’ responses have a good fit.

### Learning Progression Verification and Modification

#### GDINA Model Analysis

The results of the GDINA model analysis show that 1624 students are classified into 34 AMPs (Table 5). All students mainly concentrated in the following six AMPs: AMP 1, 4, 11, 13, 31, and 34.

**TABLE 5:** Classification of students’ AMPs.

	AMP	Total (%)	Grade 7 (%)	Grade 8 (%)	Grade 9 (%)	Grade 10 (%)	Grade 11 (%)
1	000000000	5.54	11.46	13.21	2.98	0.00	0.00
2	100000000	1.29	1.86	4.20	0.33	0.00	0.00
3	100000010	0.06	0.31	0.00	0.00	0.00	0.00
4	110000000	4.86	8.36	14.11	1.32	0.28	0.00
5	110000011	0.06	0.00	0.30	0.00	0.00	0.00
6	110001000	2.22	4.64	5.11	0.99	0.28	0.00
7	110001010	0.12	0.00	0.60	0.00	0.00	0.00
8	110001011	0.62	1.24	1.20	0.33	0.28	0.00
9	110100000	2.65	3.41	6.61	2.98	0.28	0.00
10	110100011	0.25	0.62	0.60	0.00	0.00	0.00
11	110101000	9.91	15.48	13.51	15.56	4.80	0.64
12	110101010	0.74	1.86	1.20	0.66	0.00	0.00
13	110101011	13.49	18.89	14.71	6.95	14.12	12.18
14	111000000	0.06	0.31	0.00	0.00	0.00	0.00
15	111001000	0.31	0.31	0.30	0.33	0.28	0.32
16	111001111	0.06	0.00	0.00	0.00	0.28	0.00
17	111010000	0.06	0.00	0.30	0.00	0.00	0.00
18	111011011	0.12	0.31	0.00	0.00	0.28	0.00
19	111100000	0.06	0.00	0.00	0.33	0.00	0.00
20	111101000	1.54	1.86	1.80	2.65	1.13	0.32
21	111101010	0.06	0.00	0.00	0.00	0.00	0.32
22	111101011	1.29	1.55	0.30	1.66	1.98	0.96
23	111101100	0.74	0.62	0.90	1.66	0.28	0.32
24	111101110	0.25	0.62	0.30	0.00	0.00	0.32
25	111101111	3.76	0.31	0.90	1.32	8.19	7.69
26	111110000	0.18	0.62	0.00	0.00	0.28	0.00
27	111110011	0.12	0.00	0.00	0.66	0.00	0.00
28	111110111	0.25	0.00	0.00	0.00	0.85	0.32
29	111111000	3.76	7.74	3.90	3.31	2.54	1.28
30	111111010	0.37	0.00	0.00	0.33	1.13	0.32
31	111111011	10.10	8.36	6.01	7.95	14.97	12.82
32	111111100	3.45	1.55	2.40	6.62	4.24	2.56
33	111111110	0.86	0.31	0.60	1.32	1.13	0.96
34	111111111	30.79	7.43	6.91	39.74	42.37	58.65

Further analysis of these AMPs reveals that 94.6% of students can develop a perception of randomness because they have mastered A1. 93.11% of students are able to list all possible outcomes of a one-dimensional event due to their proficiency in A1 and A2. 84.62% of students can calculate the probability of a one-dimensional event, in view of their mastery of A1, A2, and A4. 84.56% of students know how to compare the probability of one-dimensional events, which stems from their mastery of A1, A2, and A6. 63.36% of students can estimate the probability of a one-dimensional event because of their mastery of A1 and A8. 61.71% of students can form good one-dimensional probabilistic thinking as they have mastered A1, A2, A4, A6, and A8.

By shifting the discussion of students’ probabilistic thinking from one-dimensional to two-dimensional, 60.91% of students can build a connection from one-dimensional to two-dimensional on the probability estimation (A8, A9), with a slightly reduced proportion of the latter. The percentage of students who can migrate from one-dimensional sample space (A2) to two-dimensional sample space (A3) drops significantly to 58.19%. The number of students able to progress from one-dimensional probability comparisons (A4) to two-dimensional probability comparisons (A5) decreases from 84.62 to 50.06%. The proportion of students who can calculate the probability of a two-dimensional event by definition (A7) is 40.16%. However, only 30.79% of students have mature probabilistic thinking (A1–A9). In summary, we can find that middle school students have basically formed a good one-dimensional probabilistic thinking, but the development of students’ two-dimensional probabilistic thinking is not optimistic.

In terms of the classification of students in each grade, students in grade 7 are mainly concentrated in AMP 1, 4, 6, 11, 13, 29, 31 and 34, which indicates that they have a good mastery of A1, A2, A4, and A6. Students in grade 8 are mainly concentrated in AMP 1, 2, 4, 6, 9, 11, 13, 31 and 34, and the AMP 34 showed that students in grade 7 had more percentage than grade 8. This phenomenon may be due to the fact that after learning probability concepts in primary school, students in grade 8 have not been exposed to probability concepts for a longer period of time than students in grade 7, so their performance is somewhat backward. Students in grade 9 are mainly concentrated in AMP 11, 13, 31, 32 and 34, indicating that they have further mastered A3, A8, and A9 on the basis of grades 7 and 8. Students in grades 10 are mainly concentrated in AMP 11, 13, 25, 31, 32 and 34, which means that they have made great progress in probability estimation (A8, A9). Students in grades 11 are mainly concentrated in AMP 13, 25, 31 and 34, which shows that they can master almost all the attributes, and the proportion of students with mature probabilistic thinking increases from 7.43 to 58.65%.

#### Learning Progression Verification and Modification Process

The attribute mastery probability, which can be estimated by GDINA model analysis, is used to verify the hypothesized learning progression. If the hypothesized learning progression can truly reflect the development of students’ probabilistic thinking, the attribute mastery probability should be directly affected by the level of the attributes in hypothesized learning progression. That is, the attributes at a higher level should be more difficult to master, while the attributes at a lower level should be easier to master.

The GDINA model analysis shows that the order of attribute mastery probability from high to low is A1 (0.94), A2 (0.93), A6 (0.85), A4 (0.84), A8 (0.63), A9 (0.61), A3 (0.58), A5 (0.50), A7 (0.41). According to this result, in the one-dimensional probability concepts, A1, A2, A4, and A6 are relatively easy to master, except for the probability estimation of a one-dimensional event (A8). Then, A3, A8, and A9 are at a moderate difficulty level. Moreover, A5 and A7 are more difficult to master. This indicates that the attribute mastery probability levels are basically consistent with the hypothesized learning progression levels. However, the attribute mastery probability of A8 at the Level 2 of the hypothesized learning progression is lower than expected.

So as to modify the hypothesized learning progression, we first determine the transition points of the hypothesized learning progression levels from the perspective of the attribute mastery probability. According to the GDINA model analysis, the mastery probability of all attributes is within the range of 0.4 to 0.95. Meanwhile, 5.5% of the students still cannot master any attributes related to probability (see Table 5). Therefore, the Level 1 of learning progression is set so that students cannot master any attributes. Next, we divide 0.4 to 0.95 into three parts, and each part corresponds to a learning progression level. Based on the perspective of attribute mastery probability, the modified learning progression is presented in Table 6.

**TABLE 6:** Modified learning progression of probability.

Level	Content	Attributes
1	Students cannot understand any attributes related to probability.	None
2	Students begin to understand the four one-dimensional probability concepts (randomness, sample space, probability of an event and probability comparisons). But they cannot perform well in probability estimation of a one-dimensional event, and they cannot transfer their understanding of probability concepts from one-dimensional to two-dimensional.	At least one of A1, A2, A4, and A6
3	Students can perform probability estimation and two-dimensional sample space. And they cannot integrate all the two-dimensional probability concepts.	Further master at least one of A3, A8, and A9
4	Students can understand two-dimensional probability comparisons and probability of a two-dimensional event. Furthermore, they can build a connection between one-dimensional probability concepts and two-dimensional probability concepts.	Further master A5 and A7

### Students’ Understanding Levels of Probability

Regarding the modified learning progression, middle school students are classified into Level 2, Level 3 and Level 4, with more students at Level 3 and Level 4. This implies that the one-dimensional probabilistic thinking of middle school students is basically mature, and the development of two-dimensional probabilistic thinking (A3, A5, A7, A9) is relatively slow, which is consistent with the three stages of the probabilistic cognitive development proposed by Piaget and Inhelder (2014).

As for the learning progression levels of students in different grades, students in grade 7 are classified into four levels on average, with more students at Level 2 and Level 3, but still 11.46% of the students cannot master any attributes. This implies that although the vast majority of students can recognize the concept of randomness, there are still a few students in the embryonic stage of probabilistic thinking, which confirms the previous research conclusions (Moritz et al., 1996; Chan, 1997; He and Gong, 2017).

Students in grade 8 are mainly classified into Level 2, and averaged at the other three levels. However, there are more students at Level 1 and Level 2 than in grade 7, suggesting that the probabilistic thinking levels of students in grade 8 are slightly degraded compared with grade 7. This may be because the teaching of probability concepts is mainly set in grade 9, while students in grade 8 have not learned the probability concepts for a long time, which leads to their backward thinking. This suggests that the teaching of probability should be properly arranged for each grade.

Students in grade 9 are mainly classified into Level 3 and Level 4, which indicates that they have basically mastered all one-dimensional probability concepts, and the two-dimensional probabilistic thinking is also developing steadily. Students in grades 10 and 11 are mainly classified into Level 4, with 42.37 and 58.65% of them reaching the AMP (111111111), which implies that about half of them have not yet formed a mature probabilistic thinking. That is to say, although students have mastered the two-dimensional sample space, they are unable to effectively establish a connection between two-dimensional probability concepts, which makes the previous studies confirmed again (Liu and Zhang, 1985; Li, 2003; He and Gong, 2017).

In short, middle school students develop a successively more sophisticated understanding of the concepts involved in the learning progression levels, but the reasons for the decline of students in grade 8 still need to be further explored.

## Discussion

The current study aimed to develop a learning progression for probability. To this end, we built a new measurement instrument based on cognitive diagnosis theory for data collection and data analysis. The findings will be discussed from the learning progression for probability, the types probability AMPs for students, learning progression verification and modification and practical implications.

### Learning Progression for Probability

The learning progression for probability, based on the cognitive diagnosis theory, is presented in Table 6. Students at Level 1 cannot master any attributes. In particular, 5.5% of the students are at Level 1, and the number of students at Level 1 decline as the grade progresses. This confirms the research conclusion of He and Gong, there are still a small number of middle school students who do not understand the concepts related to probability (He and Gong, 2017). Thus, although the curriculum of junior high school should be spiraling upward in primary school, those students whose probabilistic thinking has not yet sprouted cannot be completely ignored.

Students at Level 2 can master at least one of one-dimensional probability concepts, with the exception of probability estimation, which combines the Uni-Structural level with the Multi-Structural level in Li’s research (Li, 2003). By contrast, contemporary students have made progress in probability, suggesting that formal teaching in the early stage of secondary school has achieved good results. It is worth noting that the probability estimation found by Li’s research is out of step with the development of other one-dimensional probability concepts (Li, 2003). This may be due to the dispersion of the probability content in the junior high school. Some suggestions are also put forward for the classroom setting, which implies that we should pay more attention to the cultivation of probability estimation thoughts, and should not ignore the importance of probability estimation as the foundation of statistics learning in the future.

Students at Level 3 can further master probability estimation of a one-dimensional event, two-dimensional sample space and probability estimation of a two-dimensional event. Logically speaking, mastering one-dimensional probability concepts is the prerequisite for continuing to learn two-dimensional probability concepts. Meanwhile, the construction of sample space is the prerequisite for probability calculation and probability comparisons. This is similar to the conclusions of earlier studies by Lecoutre, Fischbein, and English that students cannot naturally understand the sample space, because the basic results in different orders should be distinguished and counted as different results (Lecoutre et al., 1990; Fischbein et al., 1991; English, 1993).

Students at Level 4 can further develop probability of a two-dimensional event and two-dimensional probability comparisons. These two attributes belong to the last stage of probabilistic cognitive development proposed by Piaget and Inhelder — the stage of formal operation, thus verifying the setting of Level 4 (Piaget and Inhelder, 2014). In discipline logic, the sample space is the basis of probability of an event and probability comparisons. However, not all students who mastered the two-dimensional sample space can enter Level 4, and the reasons are worth exploring. Referring to the answers of these students, some students have not formed a stable understanding of the sample space and are in a wandering stage and some students have a lack of calculation formula or calculation ability in the process.

### The Types Probability AMPs for Students

There are 34 AMPs for students based on the GDINA model analysis. As can be seen from Table 6, students’ AMPs for probability can be summarized into two types through the correspondence between learning progression levels and AMPs. The first type of AMPs is to master all the attributes at a lower level and then develop the attributes at the next level, such students account for 86.89% of the total. For instance, AMP 12 (110101010) indicates that students in this category have mastered A1, A2, A4, A6, and A8, that is, after mastering all the attributes at Level 2, they have developed A8 at Level 3.

It is worth noting that there is another type of AMPs. After developing partial attributes at a low level, students develop the attributes at the next level, reciprocating cycles, and finally forming mature probabilistic thinking. Such students account for 13.11% of the total, mainly at Level 3 and Level 4. Looking at the attribute hierarchy (see Figure 1), because the probability estimation A8 and A9 are independent of the other attributes, these two attributes may be the hardest for some students to master. For example, AMP 32 (111111100) indicates that students in this category have mastered A1–A7, but have not mastered A8 and A9, that is, they have mastered all attributes of Level 2 and Level 4, but for Level 3, they have only mastered A3, and we believe that they have reached Level 4.

To sum up, most students develop probabilistic thinking in a spiraling manner, while a few develop probabilistic thinking by learning each knowledge point independently. This result indicates that the curriculum, teaching and evaluation should attach importance to the cultivation and investigation of core knowledge and ability, and further thinking is still needed on how to form a good cognitive structure for students around core knowledge.

### Learning Progression Verification and Modification

As the levels of learning progression correspond to the attributes contained in probability, the results of GDINA model analysis are used to verify and modify the hypothesized learning progression. On the one hand, the order of attribute mastery probability is basically consistent with the levels of hypothesized learning progression, that is, the attributes at a low level are easier to master, while the attributes at a high level are more difficult to master. However, the attribute mastery probability of probability estimation of a one-dimensional event is lower than expected, which implies that students who can perform well on other one-dimensional probability concepts (A1, A2, A4, and A6) still perform poorly on probability estimation of a one-dimensional event (A8). On the other hand, the AMPs of each grade students can also be used to verify and modify the hypothesized learning progression. The AMPs of students in grades 7 and 8 show that they can perform well in A1, A2, A4 and A6, and slightly worse on A8. The AMPs of students in grade 9 indicate that they have further mastered A3, A8, and A9 on the basis of grades 7 and 8. Students in grades 10 and 11 can master almost all the attributes. The above analysis means that students’ understanding of all one-dimensional probability concepts is not completely synchronized in junior high school.

From the attribute hierarchy, the probability estimation of a one-dimensional event (A8), which is an approximation of the probability of an event from the experimental perspective, is independent of A2–A7. Therefore, A8 may be more difficult to master than A1, A2, A4, and A6. This finding may be due to the fact that students in the second learning phase (grades 4 to 6) have already begun the initial study of probability, but the curriculum standards and textbooks for this phase focus on one-dimensional probability concepts and do not formally introduce probability estimation. It is not until the third learning phase (grade 9) that students begin to systematically contact the idea of probability estimation. This result shows that it is unreasonable to put A8 at the Level 2 and adjust it to the Level 3.

Compared with the hypothesized learning progression, the modified learning progression has obvious advantages. From the macro perspective, the modified learning progression combines the experience of subject experts, front-line teachers, and the students’ actual learning conditions, which is closer to the development characteristics of students’ probabilistic thinking in each grade. From the micro perspective, each student’s path from a lower level to a higher level is not unique. Starting from the student’s current AMP and taking AMP 34 (111111111) as the learning target, a path can be selected to match the learning progression and attribute hierarchy. This suggests that the learning progression constructed by the GDINA model includes both macroscopic and microscopic observations, which can improve the theoretical nature of teaching decision-making, enhance the operability of teaching practice, and provide possibility for students’ self-improvement, so as to promote the integration of curriculum, teaching and evaluation.

### Practical Implications

Through the GDINA model analysis, this study used cross-sectional data to construct a learning progression for probability. Although no longitudinal data has been collected for verification, the attribute hierarchy, learning progression and the student’s AMP can still be helpful to front-line teachers. Before teaching, the results of this study can provide a more scientific analysis of learning situations for teaching design. After teaching, the cognitive diagnostic test in this study can be used to check the learning effect of students, thereby providing a plan for teaching review and teaching remedy.

Many researchers pointed out that students’ learning pathways are not unique (Baroody et al., 2004), and teachers can find several remedy pathways for students with specific AMP to master all attributes by combining the learning progression and attribute hierarchy. In addition, teachers can gather students with specific AMP, which is more effective. For example, for students with AMP (110100000), the remedy pathway may be A8→A9→A6→A3→A5→A7 or A6→A8→A9→A3→A5→A7. In the first remedy pathway, students will first learn probability estimation, then learn probability of a one-dimensional event, and finally learn the sample space, probability comparison, and probability of a two-dimensional event. In the second remedy pathway, students will first develop a good one-dimensional probabilistic thinking, and then gradually develop a mature two-dimensional probabilistic thinking.

Furthermore, a student’s individual diagnostic report, including individual test score, IRP, AMP and non-mastered attributes, can be used to conduct an in-depth analysis of the student’s knowledge state and provide personalized remedial suggestions. For example, Table 7 shows the individual information of three students. Even if they have the same score, they may have different IRPs, AMPs, and remedy pathways. This directly demonstrates the significant advantages of using cognitive diagnosis assessment to develop learning progression.

**TABLE 7:** Individual information of three students.

ID	Score	AMP	IRP	Non-mastered attributes and remedy pathway
67	14	110101000	11111111111110000100000000	A3, A5, A7, A8, A9
				A8→A9→A3→A5→A7
179	14	110101011	11011110111110000000001101	A3, A5, A7
				A3→A5→A7
529	14	110100011	11111111110000000000001111	A3, A5, A6, A7
				A6→A3→A5→A7

### Limitations and Future Work

Although this study has the above findings and implications, there are still some limitations. First, this study used cross-sectional data to construct a learning progression for probability, but learning progression itself is a developmental concept, so longitudinal data can be collected for more in-depth exploration in the future. In addition, some scholars have recently explored longitudinal cognitive diagnosis theory (Li et al., 2016; Zhan et al., 2019; Zhan, 2020), so longitudinal tracking data can be collected under the guidance of longitudinal cognitive diagnosis theory to build learning progression that can reveal more about the laws of education. Second, the effect of the constructed learning progression is not fully explored in this study, so that future research can use remedy pathways to examine the validity of the cognitive diagnosis results. For example, students can be divided into an experimental group and a control group. Courses and teaching are arranged for the students in the experimental group according to the learning progression, while the students in the control group follow the normal teaching plan. If there is a significant difference in performance between the two groups at the end of the course, we believe that the learning progression is effective. Further exploration can group students with a specific AMP and select different remedy pathways to find the most effective way for these students. In addition, realizing the computerization of students’ diagnostic reports and targeted remedial suggestions is also the direction of future development. That is, computer programs need to be programmed to report results automatically, which can help students achieve self-remedy learning.

## Data Availability Statement

The raw data supporting the conclusions of this article will be made available by the authors, without undue reservation.

## Ethics Statement

The studies involving human participants were reviewed and approved by Northeast Normal University. The participants provided their written informed consent to participate in this study.

## Author Contributions

SB participated in the design of the study, test preparation, data collection and analysis, analysis of the results, and writing the manuscript, and agreed to submit the final version of the manuscript.

## Conflict of Interest

The author declares that the research was conducted in the absence of any commercial or financial relationships that could be construed as a potential conflict of interest.
